# Main-chain programming of poly(aryl ether ketone)s enables superior high-temperature dielectric energy storage

**DOI:** 10.1039/d6sc06787e

**Published:** 2026-09-22

**Authors:** RunZhu Liu, Dongyang Chen, Yan Song, Honghong Gong, Yunchuan Xie, Guanxiang Zhang, Xiao Zhang, Yijin He, Zhicheng Zhang

**Affiliations:** a National Innovation Platform (Center) for Industry-Education Integration of Energy Storage Technology, National Key Laboratory for High Energy Pulsed Power, School of Chemistry, Xi'an Jiaotong University Xi'an 710049 P. R. China zhichengzhang@mail.xjtu.edu.cn; b National Key Laboratory of Science and Technology on Vessel Integrated Power System, Naval University of Engineering Wuhan 430033 P. R. China; c Lanzhou Petrochemical Corporation, China National Petroleum Corporation (CNPC) Lanzhou 730060 P. R. China

## Abstract

The urgent demand for dielectric films capable of operating under high temperatures and high electric fields in advanced equipment, such as motor controllers for new energy vehicles, has placed increasing requirements on high-temperature polymer dielectrics. To address the increased leakage current and consequent deterioration in energy-storage efficiency of conventional poly(aryl ether ketone) (PAEK) at elevated temperatures, a synergistic molecular design strategy based on modular backbone programming is proposed. By coordinately regulating the conjugation degree of the polymer chains, dipolar response, and chemical crosslinking behavior, PAEK-based films combining low dielectric loss with high breakdown strength are constructed. The optimized film delivers a discharged energy density of 6.94 J cm^−3^ with a charge–discharge efficiency of 90% at 150 °C, while exhibiting fast discharge characteristics, excellent long-term cycling stability, and pronounced electrical self-healing capability. This work provides a new molecular design strategy for high-temperature polymer dielectrics toward highly reliable power electronics and pulsed energy-storage devices.

## Abstract 2

Main-chain programming of PAEK integrates rigid, polar, and crosslinkable units to decouple dielectric polarization from charge transport, achieving reliable high-temperature energy storage with suppressed loss and enhanced breakdown strength.

## Graphical abstract

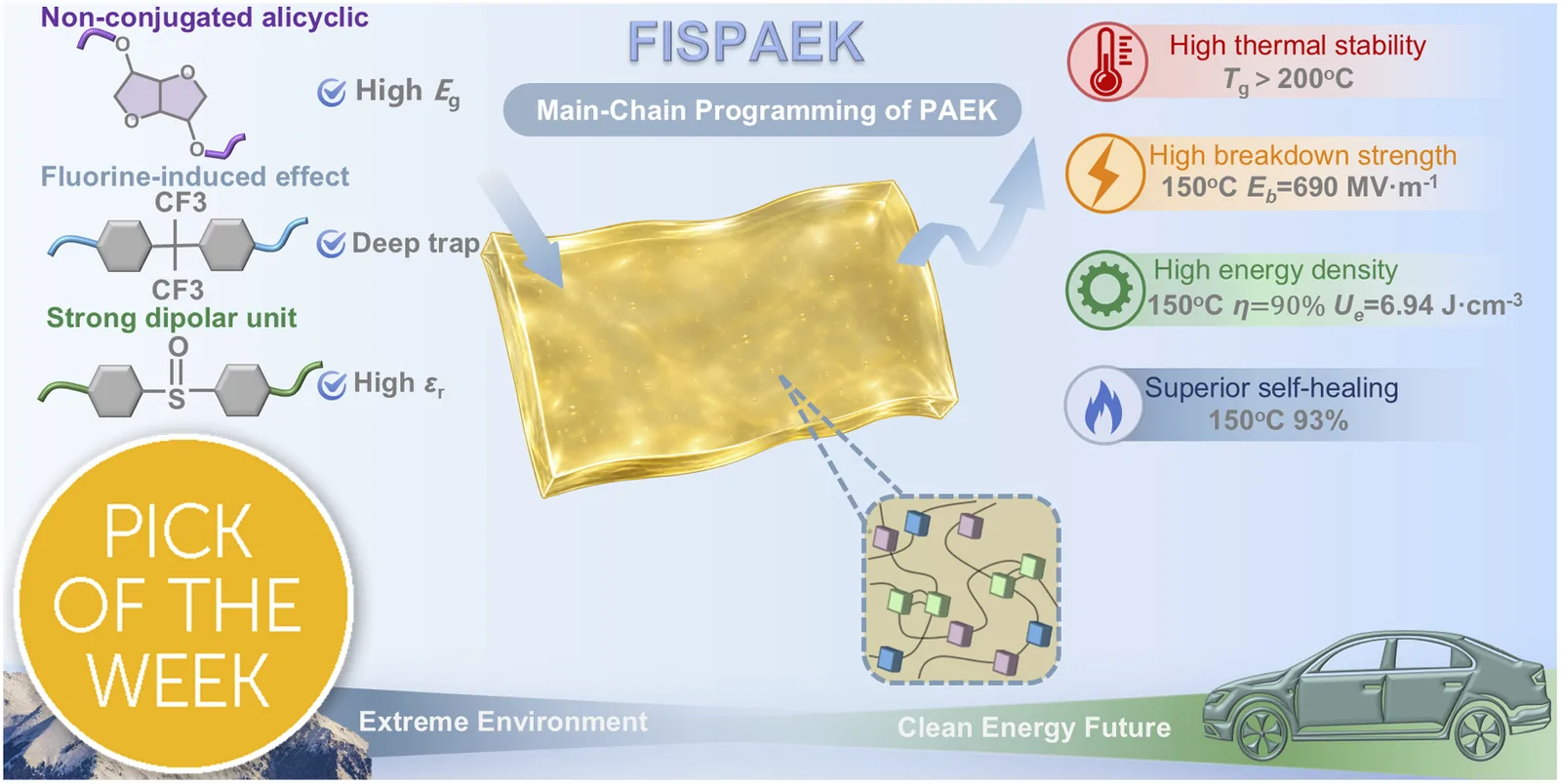

## Introduction

The rapid evolution of advanced technologies, including motor controllers for new energy vehicles, solid-state pulsed-power systems, and electromagnetic launch systems, toward operation at elevated temperatures, high frequencies, and high electric fields has created an increasing demand for high-performance dielectric films capable of stable operation at 150 °C. However, existing dielectric materials still face difficulties in simultaneously achieving thermal resistance, low loss, and high energy-storage capability.^1,2^ Commercial polyimide (PI) films suffer from substantial deterioration in discharge energy density (*U*_e_) and charge–discharge efficiency (*η*) at elevated temperatures,^2,3^ whereas biaxially oriented polypropylene (BOPP), despite its intrinsically low dielectric loss, is limited by an upper operating temperature of ≈105 °C.^4,5^ Fluorinated polar polymers can provide relatively high polarization, but their high leakage current and insufficient breakdown strength often restrict their use under harsh operating conditions.^6,7^ These limitations remain major obstacles to the further development of high-power-density film capacitors and highly reliable power electronic devices.^8^

Poly(aryl ether ketone) (PAEK) has been widely considered a promising candidate for high-temperature polymer dielectrics because of its excellent thermal resistance, mechanical strength, and thermoplastic processability.^9,10^ Nevertheless, under high-temperature and high-field conditions, PAEK remains susceptible to segmental thermal relaxation, space-charge accumulation, and local electric-field distortion, accompanied by increased dielectric loss (tan *δ*), premature breakdown, and reduced *η*.^11–13^ Current molecular modification strategies mainly rely on regulating individual structural features. One approach introduces rigid units such as biphenyl, naphthalene, or fluorene moieties to increase the energy barrier for segmental motion and improve thermal stability, but this can be accompanied by increased brittleness and limited dielectric breakdown performance.^14–16^ Another approach incorporates fluorinated groups such as –CF_3_ to regulate the electronic distribution and reduce leakage current or the dielectric constant. However, the associated steric hindrance and free-volume effects may weaken dense chain packing, reduce polarization response, and potentially introduce additional charge-transport defects.^17,18^ Likewise, crosslinking can restrict segmental motion and suppress high-temperature loss, but may compromise dielectric response or processability.^19^ Thus, most existing approaches improve only selected aspects of dielectric performance and remain insufficient to simultaneously achieve intrachain conjugation decoupling, polarization regulation, and high-temperature stability within a single molecular framework.^20–23^ A molecular programming strategy that integrates multiple functional motifs into one PAEK backbone is therefore highly desirable for the concurrent improvement of thermal resistance, low-loss characteristics, and breakdown strength (*E*_b_).

Here, a modular backbone programming strategy is developed by integrating saturated alicyclic conjugation-decoupling units, fluorinated electronic-regulation units, and polar crosslinkable units into the PAEK backbone. The conjugation characteristics, dipolar response, and chemical crosslinking behavior of the polymer chains are thereby coordinately regulated to construct PAEK films with low tan *δ* and high *E*_b_ at elevated temperatures. The resulting FISPAEK retains a favorable dielectric response while exhibiting suppressed high-temperature leakage conduction and enhanced *E*_b_. It further retains high *U*_e_ and high *η* at 150 °C, together with fast discharge behavior, long-term cycling stability, and electrical self-healing capability. This work provides a molecular design strategy for the multi-objective optimization of high-temperature polymer dielectrics.

## Results and discussion

### Molecular design and property simulation

To mitigate the electronic delocalization and high-temperature charge transport associated with the continuous aromatic structure of conventional PAEK, a modular molecular programming strategy was developed by integrating saturated alicyclic decoupling units, fluorinated electronic-regulation units, and polar crosslinkable units into the PAEK backbone. This design enables molecular-level regulation of orbital delocalization, dipolar response, and the local electrostatic environment. The synthetic route to FISPAEK is shown in Fig. 1a. To clarify the intrinsic electronic characteristics of the individual functional modules, the dipolar polarization energy (DPE), bandgap (*E*_g_), and orbital delocalization index (ODI) of isosorbide (IS), hexafluorobisphenol A (6F), and 4,4′-sulfonylbis(2-allylphenol) (DAS) were first calculated.^24,25^ IS exhibits the largest *E*_g_ and the highest ODI, indicating a wide intrinsic energy-level separation and a strong tendency toward orbital localization, and thus serves as a saturated decoupling unit for limiting long-range electronic delocalization. DAS shows the highest DPE, reflecting a strong intrinsic dipolar response, while also providing reactive sites for subsequent thermal crosslinking. The descriptors of 6F fall within a relatively balanced range, while the strong electron-withdrawing effect and steric hindrance of its fluorinated groups provide a structural basis for regulating the electronic distribution along the backbone and interchain interactions.

**Fig. 1:**
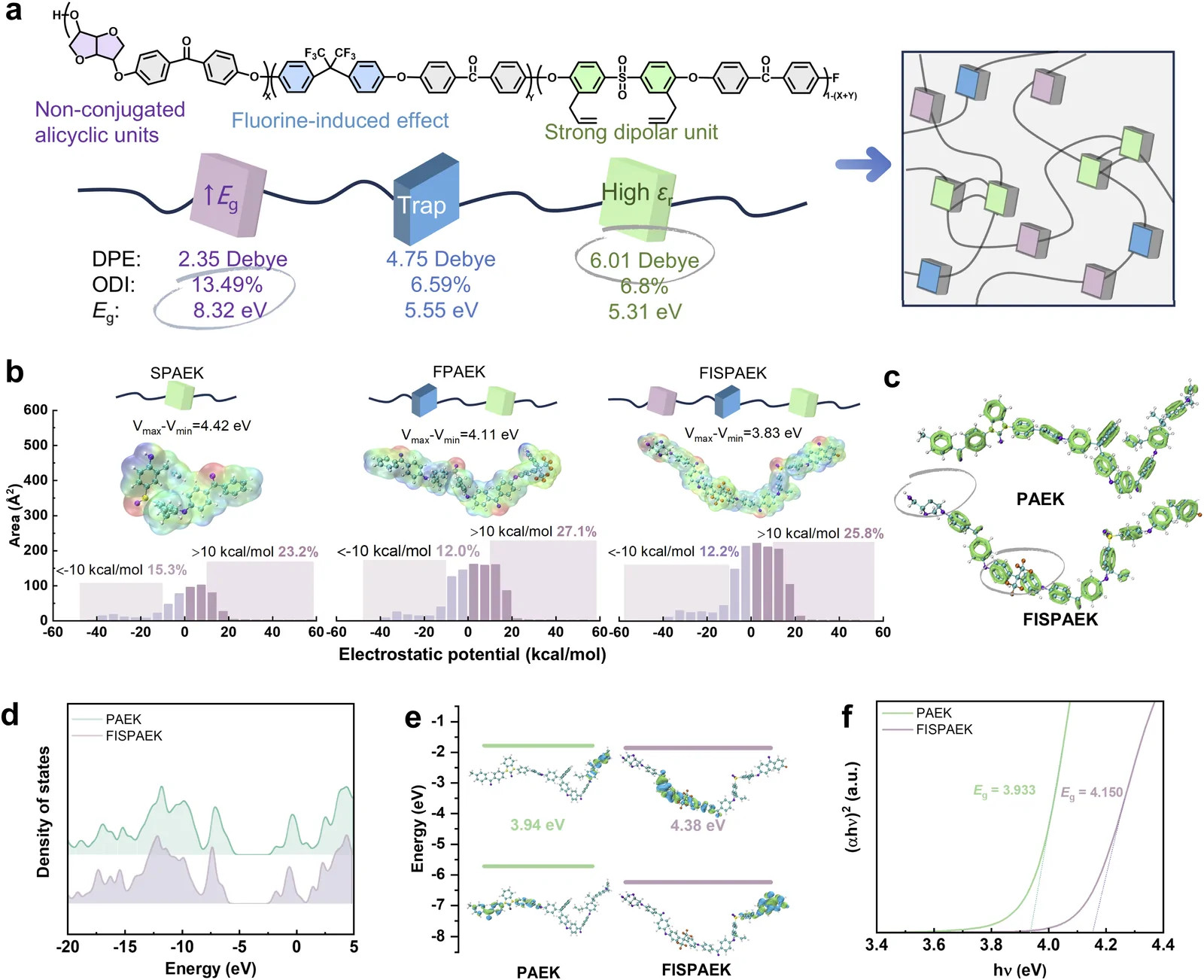
Structural design of FISPAEK. (a) Synthetic route of FISPAEK. (b) Surface electrostatic potential (ESP) distributions, corresponding surface-area fractions, and potential extrema differences (*V*_max_ − *V*_min_) of SPAEK, FPAEK, and FISPAEK. (c) π-Electron density distributions of PAEK and FISPAEK. (d) Density of states (DOS) of PAEK and FISPAEK. (e) HOMO and LUMO distributions of PAEK and FISPAEK. (f) Tauc plots and optical bandgaps of PAEK and FISPAEK films derived from UV-Vis spectra.

The roles of the incorporated functional units were further evaluated using SPAEK, FPAEK, and FISPAEK as a stepwise model series. SPAEK contains DAS, FPAEK incorporates 6F together with DAS, and FISPAEK further introduces IS into the 6F/DAS-containing backbone. As shown by the three-dimensional surface electrostatic potential (ESP) maps and corresponding surface-area distributions in Fig. 1b, the incorporation of 6F in FPAEK reduces the areas associated with extreme positive and negative potentials and decreases *V*_max_ − *V*_min_ relative to SPAEK, indicating weakened local electrostatic heterogeneity. With the further introduction of IS, the negative-potential area remains nearly unchanged, whereas the positive-potential regions are redistributed and *V*_max_ − *V*_min_ decreases further in FISPAEK, resulting in a narrower overall surface-potential distribution. In parallel, disruption of intrachain π-electron continuity provides another means of limiting electronic delocalization. As shown in Fig. 1c, the π-electron cloud extends continuously along the aromatic backbone of PAEK, whereas in FISPAEK the continuous π-electron distribution does not extend across the hexafluoroisopropylidene linkage or the isosorbide unit, indicating suppressed long-range π delocalization.

The density of states (DOS) and HOMO–LUMO distributions in Fig. 1d and e further show that incorporation of the functional units modifies the distributions of occupied and unoccupied electronic states and widens the frontier-orbital energy separation of FISPAEK relative to PAEK. The Tauc plots and optical *E*_g_ results in Fig. 1f follow the same trend as the theoretical calculations, providing experimental support for the regulation of the backbone electronic structure by modular incorporation. Collectively, IS, 6F, and DAS contribute to the molecular design through orbital localization, electronic-distribution regulation, dipolar response, and crosslinking functionality, respectively. These structural and electronic characteristics provide a molecular basis for balancing polarization response and electrical insulation in FISPAEK and for the subsequent suppression of high-temperature leakage conduction and dielectric loss.

### Synthesis, structural characterization, and thermomechanical properties of FISPAEK

The theoretical results above suggest that the cooperative incorporation of different functional units may improve the dielectric and energy-storage performance of FISPAEK. To examine this prediction experimentally, PAEK and FISPAEK were synthesized by nucleophilic polycondensation, with PAEK prepared according to our previously reported procedure.^26^ The side-chain allyl groups provide reactive sites for subsequent construction of a flexible crosslinked network. During annealing of the solution-cast films at 200 °C, linear FISPAEK undergoes further crosslinking through free-radical addition reactions. The detailed synthesis and crosslinking processes are shown in Fig. S1. The chemical structures of PAEK and FISPAEK were characterized by ^1^H nuclear magnetic resonance (^1^H NMR) spectroscopy and Fourier transform infrared (FTIR) spectroscopy, respectively, as shown in Fig. 2a and b. The results confirm the successful formation of the target structures, while the molecular weights of PAEK and FISPAEK are provided in Fig. S2. Notably, the annealed films remained insoluble in DMAc after storage for 60 days (Fig. S3), indicating the formation of a stable crosslinked network. Surface scanning electron microscopy (SEM) images and the corresponding elemental mapping show a uniform distribution of the constituent elements throughout the crosslinked films (Fig. S4), demonstrating good compositional homogeneity. The dense and smooth cross-sectional morphology in Fig. S5 further confirms the formation of compact crosslinked films.

**Fig. 2:**
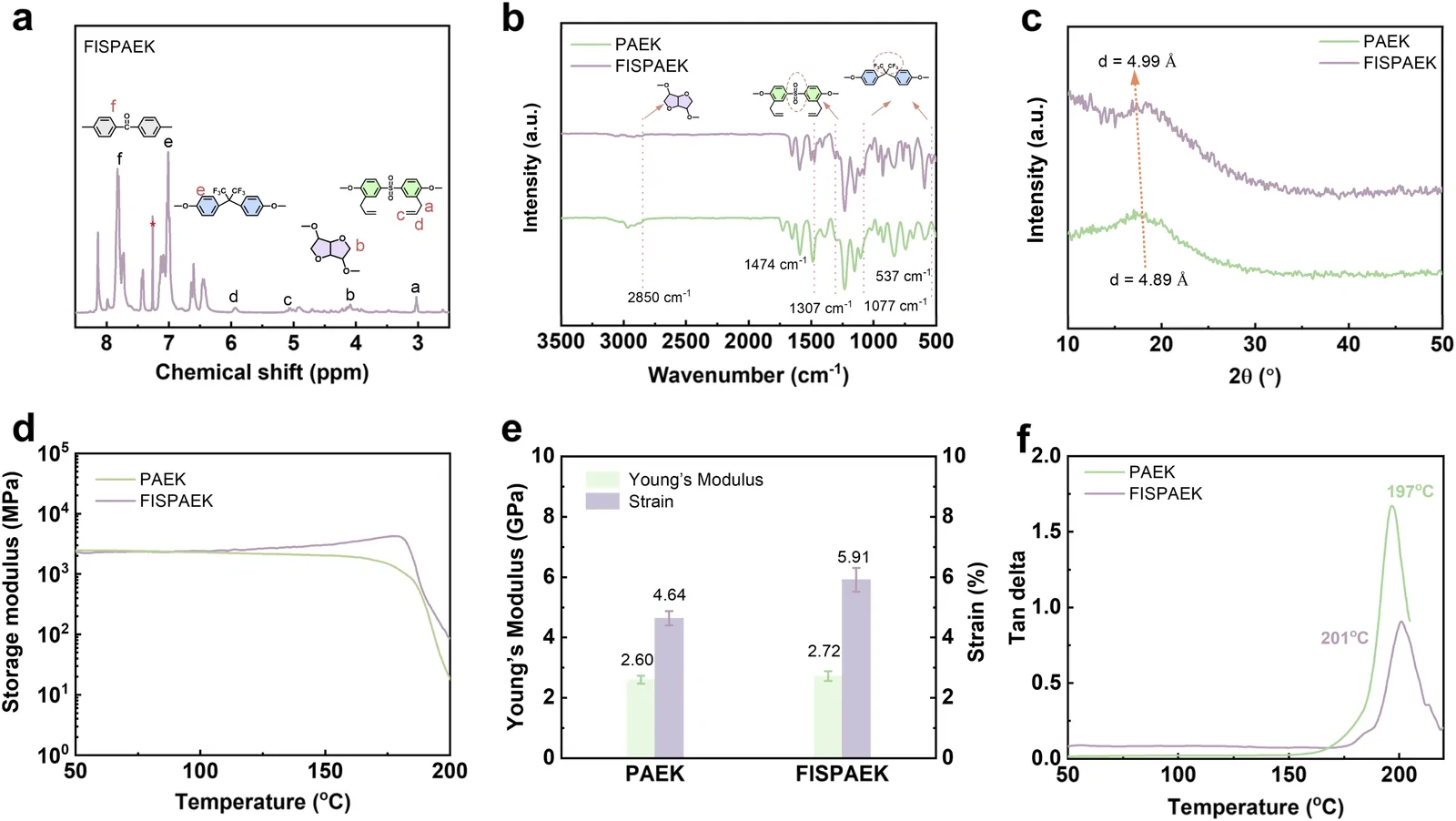
Structural characterization and thermomechanical properties of FISPAEK. (a) ^1^H NMR spectrum of FISPAEK. (b) FT-IR spectra of PAEK and FISPAEK. (c) X-ray diffraction (XRD) patterns and corresponding interchain distances of PAEK and FISPAEK. (d) Storage moduli of PAEK and FISPAEK films measured by dynamic mechanical analysis (DMA). (e) Young's modulus and elongation at break of PAEK and FISPAEK films. (f) Glass transition temperatures (*T*_g_) of PAEK and FISPAEK films determined from DMA measurements.

The structural regulation is further reflected in the condensed-state organization and macroscopic properties. As shown by the XRD results in Fig. 2c, the characteristic diffraction peak of FISPAEK shifts toward a lower angle, indicating an increased interchain distance. The incorporation of saturated alicyclic decoupling units, fluorinated electronic-regulation units, and polar crosslinkable units leads to looser molecular-chain packing. The resulting reduction in interchain dipolar coupling and intermolecular interactions is favorable for suppressing dielectric loss at elevated temperatures. Meanwhile, the crosslinked structure improves the mechanical stability required for operation under high electric fields. The gel fraction results in Fig. S6 show that FISPAEK has a high degree of crosslinking, indicating that a denser three-dimensional network can be formed through the side-chain allyl sites. During thermal treatment, multiple reactive sites along the polymer chains participate in crosslinking, producing a more complete network and effectively restricting large-scale segmental motion.

Accordingly, FISPAEK exhibits a pronounced increase in the storage modulus (Fig. 2d), together with an increased Young's modulus (Fig. 2e), indicating that the crosslinked network enhances resistance to mechanical deformation. At the same time, the flexible network formed by crosslinking of the side-chain allyl groups, together with the increased interchain distance, provides greater cooperative deformability. The elongation at break therefore increases to some extent (Fig. S7), while the film-winding behavior in Fig. S8 further demonstrates the flexibility of the film. In terms of thermal properties, FISPAEK exhibits a glass transition temperature of 201 °C. Meanwhile, the tan *δ* peak in the DMA curve is markedly weakened, indicating reduced energy dissipation during segmental relaxation (Fig. 2f). Combined with the increased interchain distance revealed by XRD, these results suggest that the functional units weaken intermolecular interactions and alter the chain-packing state, thereby reducing relaxation loss under thermal excitation.

Overall, through the synergistic effects of chain-structure regulation, condensed-state optimization, and crosslinked-network construction, FISPAEK achieves structural stabilization from the molecular to the macroscopic scale, providing a solid foundation for its excellent high-temperature insulation and energy-storage performance.

### Dielectric and breakdown properties of FISPAEK and the underlying mechanisms

The molecular programming strategy further enables coordinated regulation of dielectric response and high field insulation performance. To clarify the compositional effects of the individual functional units, a series of FISPAEK samples with different compositions were prepared, including three control samples enriched in IS, DAS, and 6F, respectively. Their compositions are summarized in Table S1. The room temperature dielectric spectra show that all FISPAEK films retain stable dielectric responses over the frequency range from 0.1 Hz to 1 MHz, as shown in Fig. 3a, S9 and S10. Increasing the DAS content enhances the dielectric constant (*ε*_r_) but also increases dielectric loss (tan *δ*), whereas enrichment of 6F reduces the tan *δ* at the expense of *ε*_r_. The optimized FISPAEK composition provides the best balance between dielectric response and loss suppression. At the molecular level, the saturated alicyclic decoupling unit acts as a nonconjugated node that disrupts continuous intrachain π conjugation and weakens electronic delocalization. The fluorinated electronic regulation unit adjusts the electron density distribution, weakens interchain electrostatic interactions, and suppresses charge diffusion and transfer. Meanwhile, the polar crosslinkable unit contributes to polarization^27^ and forms a stable network that restricts segmental relaxation and carrier migration. As a result, FISPAEK retains a tan *δ* value below 0.005 while retaining a dielectric constant higher than that of conventional PAEK.^13,16^

**Fig. 3:**
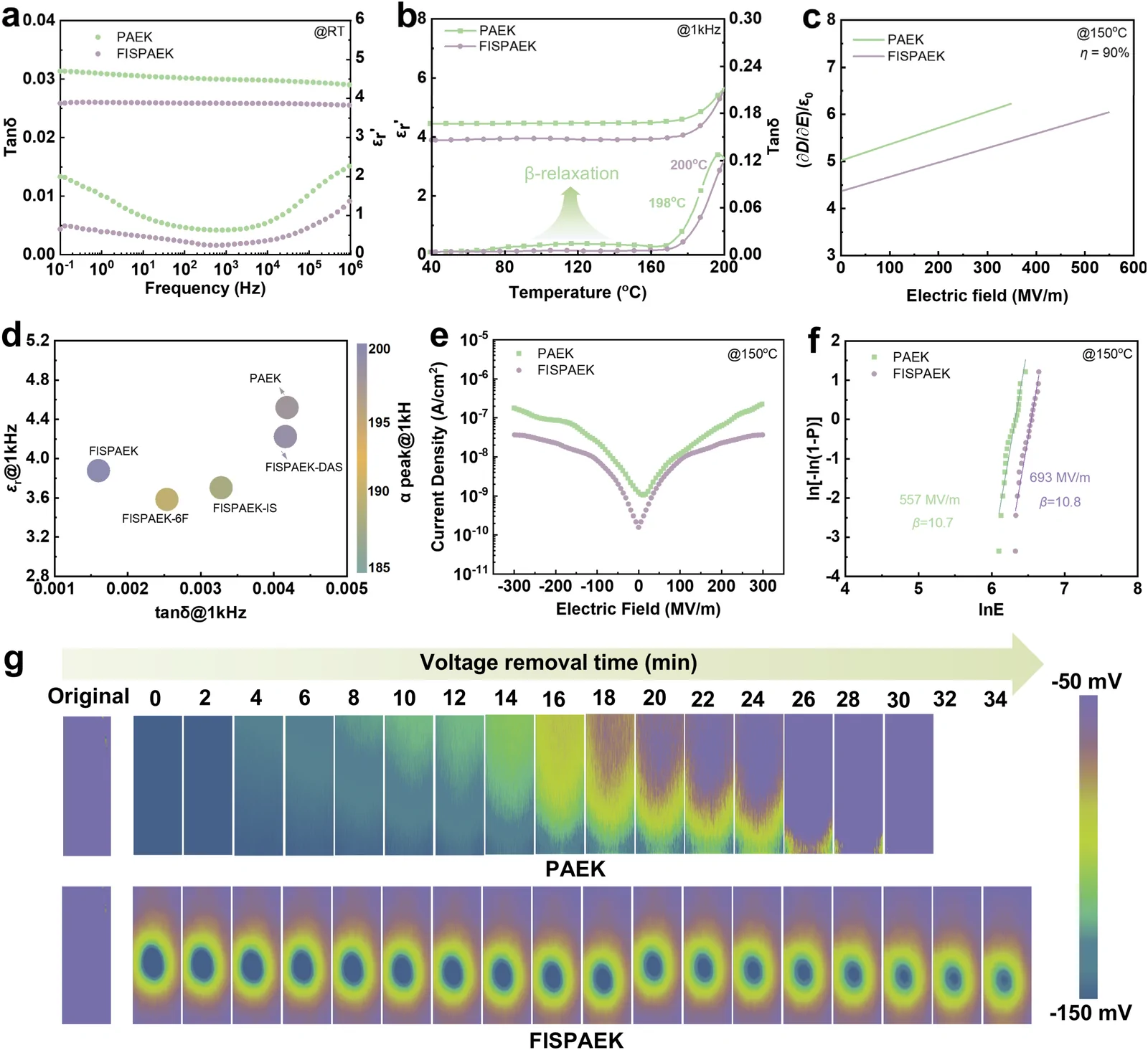
Dielectric and breakdown properties of FISPAEK. (a) Frequency-dependent *ε*_r_ and tan *δ* of different PAEKs at room temperature. (b) Temperature-dependent *ε*_r_ and tan *δ* of different PAEKs at 1 kHz. (c) (∂*D*/∂*E*)/*ε*_0_ of PAEK and FISPAEK at *η* = 90% and 150 °C. (d) Comprehensive dielectric-performance map of FISPAEK films with different compositions, evaluating room-temperature dielectric properties, high-temperature dielectric properties, and the overall performance score. The detailed compositions are listed in Table S1. (e) Leakage current density of PAEK and FISPAEK at 150 °C. (f) Weibull breakdown distributions of PAEK and FISPAEK at 150 °C. (g) Surface-potential decay of FISPAEK measured by kelvin probe force microscopy (KPFM).

The temperature dependent dielectric behavior further demonstrates the thermal stability of FISPAEK, as shown in Fig. 3b, S11 and S12. Compared with PAEK, FISPAEK exhibits a more stable *ε*_r_ value and lower tan *δ* with increasing temperature. Its *α* relaxation peak is markedly weakened, while the low temperature *β* relaxation is also suppressed. This behavior is attributed to the combined roles of the three functional units. The thermally crosslinked network formed by DAS restricts cooperative main chain motion, the rigid IS structure suppresses local conformational transitions, and the steric hindrance introduced by 6F further impedes segmental rearrangement. Together, these effects suppress the rapid increase in free volume with temperature and delay thermally activated relaxation and orientation of polar groups, thereby reducing relaxation loss at elevated temperatures. At 150 °C and under a high electric field corresponding to *η* = 90%, FISPAEK shows a more stable linear *D*–*E* response, further indicating reduced loss (Fig. 3c). Fig. 3d provides an overall comparison of room temperature dielectric properties, high-temperature stability, and *α* relaxation behavior. FISPAEK exhibits the best balance among these performance metrics and was therefore selected as the optimized composition for subsequent high field studies.

At 150 °C, the leakage current density of FISPAEK is significantly lower than that of PAEK, indicating effective suppression of high-temperature carrier transport (Fig. 3e). Correspondingly, its characteristic Weibull breakdown strength increases from approximately 557 MV m^−1^ for PAEK to approximately 693 MV m^−1^ for FISPAEK (Fig. 3f). KPFM measurements further reveal a slower surface potential decay for FISPAEK (Fig. 3g), indicating stronger trapping and confinement of injected charges. The electronic regulation introduced by the fluorinated units, local traps associated with the polar groups, and restriction of segmental motion by the crosslinked network collectively reduce charge migration and local electrothermal instability. These effects contribute to the concurrent reduction in dielectric loss and enhancement of high-temperature breakdown strength.^28–30^

### Energy-storage performance of FISPAEK

The molecular programming strategy not only improves the dielectric response and breakdown behavior but also translates into enhanced energy-storage performance. The polarization behavior of FISPAEK films under high electric fields was evaluated using *D*–*E* loops, as shown in Fig. S13. Both PAEK and FISPAEK exhibit typical linear dielectric characteristics. The *U*_e_ and *η* were calculated from the *D*–*E* loops according to
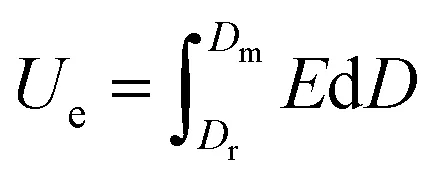
and
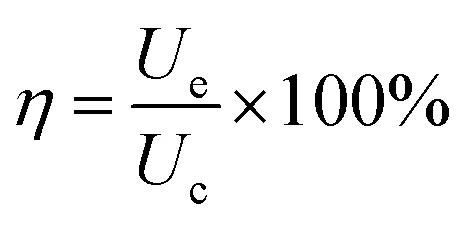
where *D*_m_ and *D*_r_ are the maximum and remnant electric displacements, respectively, and *U*_c_ is the charged energy density. For an approximately linear dielectric, the energy-storage capability can be described by
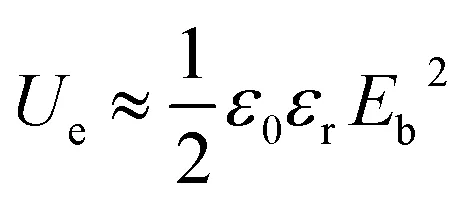
where *ε*_0_, *ε*_r_, and *E*_b_ denote the vacuum permittivity, dielectric constant, and breakdown strength, respectively.^13^

As shown in Fig. 4a–c, FISPAEK delivers a discharged energy density of 12.73 J cm^−3^ at room temperature with an *η* of 90%, representing an increase of ≈118% over PAEK. At 150 °C, it still achieves 6.94 J cm^−3^ with an *η* of 90%, corresponding to an improvement of ≈121% over PAEK. This enhancement is attributed to the reduced loss and increased breakdown strength arising from the combined effects of the saturated alicyclic decoupling units, fluorinated electronic-regulation units, and polar crosslinkable units. Fig. 4d and Table S2 further compare the *U*_e_ and *η* of FISPAEK with those of reported commercial energy-storage polymers and recently reported polymer composites at 150 °C. FISPAEK combines a relatively high *U*_e_ with high *η* and shows competitive overall energy-storage performance at elevated temperatures.

**Fig. 4:**
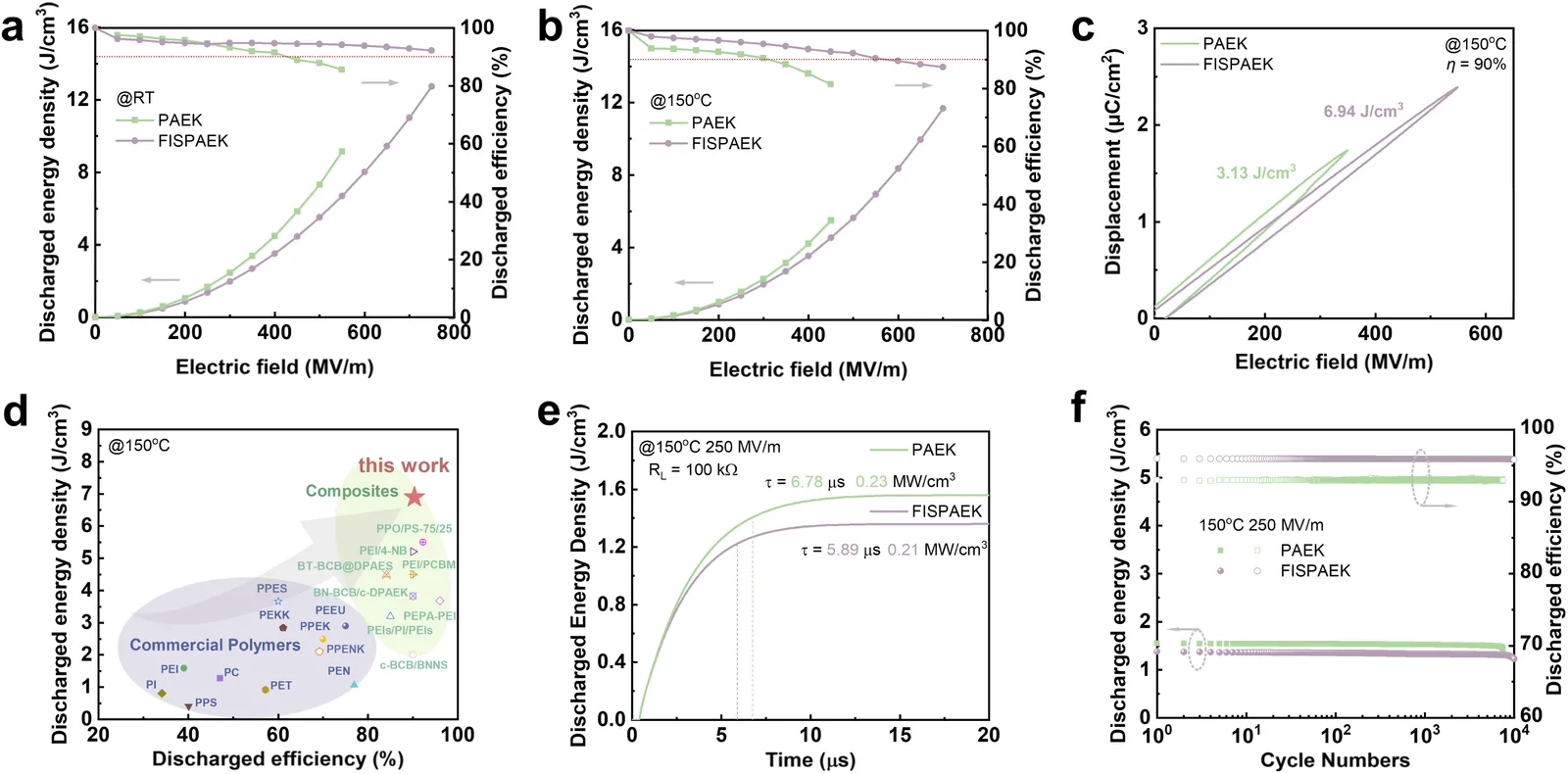
Capacitive energy-storage performance of FISPAEK. Discharged energy density (*U*_e_) and charge–discharge efficiency (*η*) of PAEK and FISPAEK (a) at room temperature and (b) 150 °C. (c) *D*–*E* loops of PAEK and FISPAEK at 150 °C with an *η* of 90%. (d) Comparison of the *U*_e_ and *η* at 150 °C between this work and other reported high-temperature dielectric polymers. (e) Fast discharge behavior of PAEK and FISPAEK films at 150 °C under an electric field of 250 MV m^−1^ and at a load resistance of 100 kΩ, presented as *U*_e_ as a function of time. (f) Cycling performance of PAEK and FISPAEK films at 150 °C and 250 MV m^−1^.

The rapid discharge performance of the film is also improved. The discharge time (*t*_0.9_), defined as the time required to release 90% of the total discharged energy, was evaluated to characterize the energy-release rate. At 150 °C and 250 MV m^−1^, FISPAEK exhibits a *t*_0.9_ of approximately 6 µs, demonstrating its rapid energy-release capability (Fig. 4e). Long-term durability is equally important for film capacitors. FISPAEK was therefore subjected to 10 000 charge–discharge cycles at 150 °C and 250 MV m^−1^. Its power density remains highly stable throughout cycling with almost no observable decay, demonstrating excellent cycling stability under prolonged high-temperature and high-field conditions (Fig. 4f). Overall, FISPAEK achieves concurrent improvements in discharged energy density, charge–discharge efficiency, power output, and cycling stability at elevated temperatures, highlighting its potential as a dielectric material for high-temperature film capacitors.^31^

### Electrical self-healing performance and mechanism of FISPAEK

In addition to improving dielectric energy-storage performance, the incorporation of saturated structural units in the molecular programming strategy also enhances the electrical self-healing capability of the films. After electrical breakdown, PAEK tends to form pronounced carbonized residues at the breakdown site, which can develop into local conductive paths. The large amount of energy released within a short time, together with the limited toughness of the film, also promotes crack propagation around the damaged region and aggravates the electrical damage. These features are evident in the post-breakdown optical image in Fig. 5a. In contrast, FISPAEK releases substantial gaseous products during breakdown, which helps dissipate local energy. No obvious carbonized residue is observed after breakdown, and the resulting holes exhibit smooth edges without visible cracks, as shown in Fig. 5b. These features indicate effective electrical self-healing of FISPAEK.

**Fig. 5:**
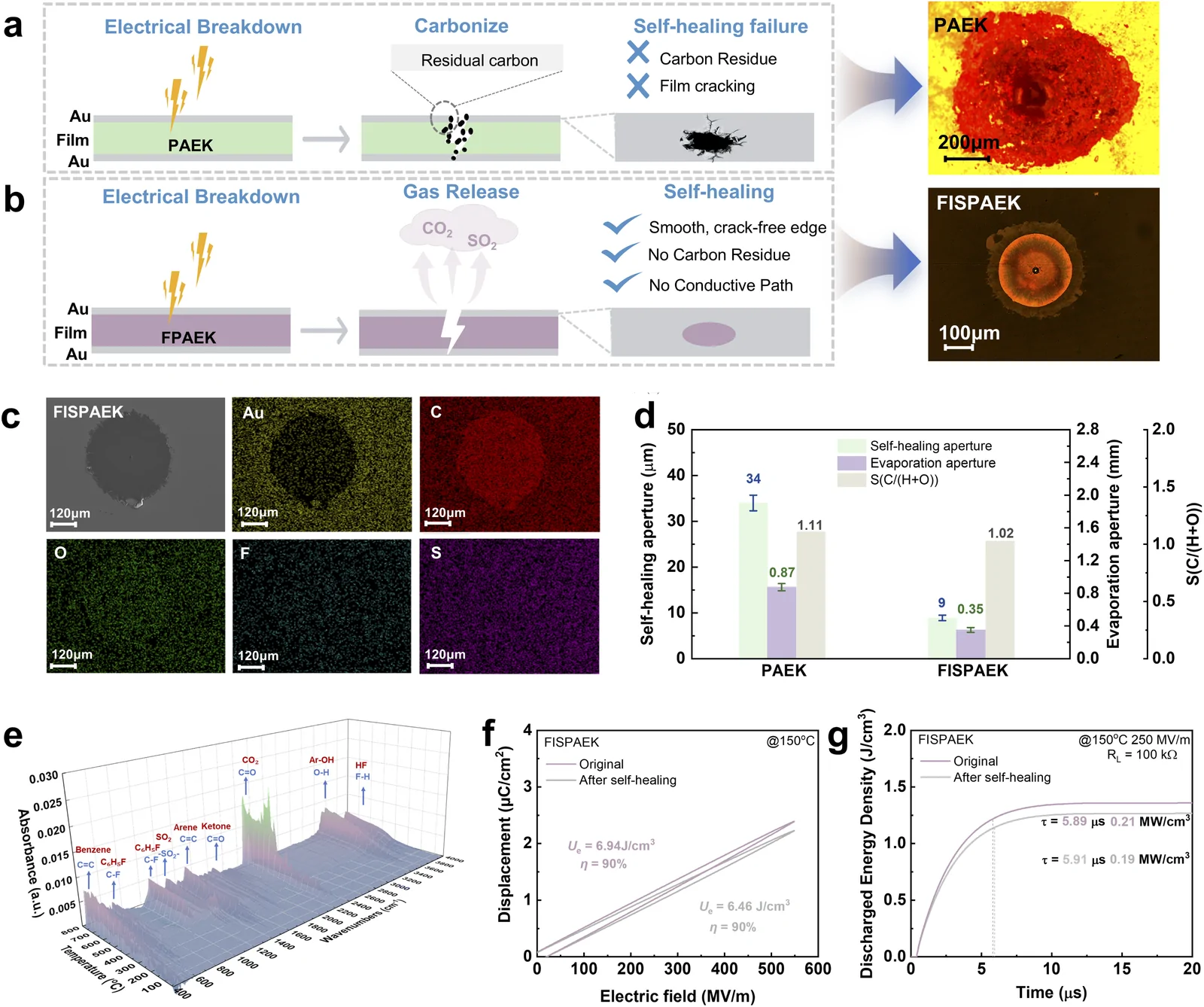
Electrical breakdown self-healing behavior of FISPAEK. Schematic illustrations of electrical breakdown and corresponding self-healing images of (a) PAEK and (b) FISPAEK. (c) SEM image and corresponding elemental mapping of the FISPAEK film after breakdown. (d) Distributions of the self-healing hole diameter and electrode-cleared area diameter, together with the carbon contents of PAEK and FISPAEK films. (e) 3D thermogravimetric infrared spectroscopy (TG-IR) spectra of the FISPAEK film. (f) *D*–*E* loops of the FISPAEK film before and after electrical self-healing. (g) Fast discharge behavior of the FISPAEK film before and after electrical breakdown self-healing at 150 °C under an electric field of 250 MV m^−1^ and at a load resistance of 100 kΩ, presented as *U*_e_ as a function of time.

The breakdown sites of FISPAEK were further examined by SEM and EDS to clarify the underlying process. As shown in Fig. 5c, no obvious carbon enrichment is detected in the damaged region, indicating that a stable residual carbonaceous conductive path is not formed locally. The breakdown-hole size of FISPAEK is also markedly reduced, as shown in Fig. 5d. This behavior is mainly attributed to the lower effective carbon content and reduced carbonization tendency resulting from the incorporation of saturated structures, which suppress local carbonization and limit damage propagation during breakdown. The 3D TG-IR spectra in Fig. 5e further show substantial gas evolution during thermal decomposition of FISPAEK, including volatile products such as CO_2_ and SO_2_. To further clarify the thermal decomposition products of FISPAEK, the characteristic absorption bands in the TG-IR spectra were assigned, and the corresponding vibrational modes and gaseous products are summarized in Table S4. Such gas release can help remove excess local heat and reduce the formation of residual conductive phases during breakdown.

The energy-storage and charge–discharge performance of FISPAEK was further evaluated after successful electrical self-healing. Only a slight degradation is observed, while the film retains high *U*_e_ and *η*, as shown in Fig. 5f, together with fast discharge capability in Fig. 5g. These results indicate that FISPAEK can effectively restore its insulation function and retain energy-storage output after local electrical damage, supporting its potential for long-term operation in highly reliable high-temperature film capacitors.^32–35^

## Conclusions

This work develops a molecular design strategy based on modular backbone programming by integrating saturated alicyclic decoupling units, fluorinated electronic regulation units, and polar crosslinkable units into the PAEK backbone. The resulting molecular architecture enables multilevel regulation of conjugation decoupling, charge transport suppression, and modulus enhancement. FISPAEK therefore combines low tan *δ*, high *E*_b_, and excellent high-temperature energy storage performance. At 150 °C, it delivers a *U*_e_ of 6.94 J cm^−3^ with a high *η* of 90%, together with fast discharge capability and long-term cycling stability. FISPAEK also exhibits pronounced electrical self-healing behavior. Effective self-healing after breakdown allows the film to retain excellent energy storage output following local electrical damage. This work provides a new molecular engineering framework for the concurrent optimization of low tan *δ*, high *E*_b_, and electrical self-healing in high-temperature polymer dielectrics.

## Author contributions

Runzhu Liu: conceptualization, methodology, investigation, validation, data curation, writing – original draft, writing – review & editing. Dongyang Chen: conceptualization, methodology, software, investigation, validation, visualization. Yan Song: conceptualization, methodology, investigation, validation. Honghong Gong: conceptualization, methodology, formal analysis, visualization, supervision, project administration, writing – review & editing. Yunchuan Xie: conceptualization, methodology, formal analysis, resources, supervision, project administration, funding acquisition, writing – original draft, writing – review & editing. Guanxiang Zhang: conceptualization, methodology, investigation, validation. Xiao Zhang: conceptualization, methodology, investigation, validation. Yijin He: conceptualization, methodology, investigation, validation. Zhicheng Zhang: conceptualization, methodology, formal analysis, resources, supervision, project administration, funding acquisition, writing – original draft, writing – review & editing.

## Conflicts of interest

There are no conflicts to declare.

## Supplementary Material

SC-OLF-D6SC06787E-s001

## Data Availability

The data supporting this article have been included as part of the supplementary information (SI). Supplementary information is available. See DOI: https://doi.org/10.1039/d6sc06787e.
