# Supplementary material for: Main-chain programming of poly(aryl ether ketone)s enables superior high-temperature dielectric energy storage
Source: Chem Sci. 2026 Sep 22. Online ahead of print. doi: 10.1039/d6sc06787e (PMC13662753; doi:10.1039/d6sc06787e)
Supplement: Supplementary file 1 [file SC-OLF-D6SC06787E-s001.pdf]

Supplementary Information (SI) for Chemical Science.
This journal is © The Royal Society of Chemistry 2026

Supporting Information

Main-Chain Programming of Poly(aryl ether ketone)s Enables Superior High-Temperature Dielectric Energy Storage

RunZhu Liu[a], Dongyang Chen[a], Yan Song[a], Honghong Gong*[a], Yunchuan Xie*[a], Guanxiang Zhang[b], Xiao Zhang[b], Yijin He[c], Zhicheng Zhang*[a]

a National Innovation Platform (Center) for Industry-Education Integration of Energy Storage Technology, National Key Laboratory for High Energy Pulsed Power, School of Chemistry, Xi'an Jiaotong University, Xi'an, P. R. China 710049.

b National Key Laboratory of Science and Technology on Vessel Integrated Power System, Naval University of Engineering, Wuhan, P. R. China 430033.

c Lanzhou Petrochemical Corporation, China National Petroleum Corporation (CNPC), Lanzhou, P. R. China 730060.

1. Experiment Section

1.1 Materials

Isosorbide, hexafluorobisphenol A, 4,4′-sulfonylbis(2-allylphenol), and 4,4′-difluorobenzophenone were purchased from Shanghai Aladdin Biochemical Technology Co., Ltd. Toluene, sulfolane, and N-methyl-2-pyrrolidone (NMP, AR grade) were obtained from Tianjin Reagent Co., Ltd. Anhydrous potassium carbonate (K_2CO_3) and toluene were supplied by Shanghai Macklin Biochemical Co., Ltd., China.

1.2 Synthesis of FISPAEK

The polycondensation reaction was carried out in a 100 mL three-neck round-bottom flask heated in a silicone oil bath and equipped with a magnetic stirrer, a water-cooled condenser, a Dean-Stark trap, and a nitrogen inlet and outlet. 4,4′-Sulfonylbis(2-allylphenol) (6.608 g, 20 mmol), 4,4′-difluorobenzophenone (6.546 g, 30 mmol), isosorbide (0.7307 g, 5 mmol), hexafluorobisphenol A (1.68115 g, 5 mmol), K_2CO_3 (4.146 g, 30 mmol), sulfolane (30 mL), and toluene (10 mL) were added to the flask. Before heating, the reaction mixture was stirred under a nitrogen atmosphere for 10 min. The mixture was then slowly heated to 135°C and refluxed for 4 h, followed by gradual heating to 150 to 155°C to remove toluene. The remaining mixture was subsequently heated at 185°C for 22 h. After the reaction, the solution was poured into deionized water containing a small amount of hydrochloric acid to precipitate the polymer as strips. The polymer was then crushed, boiled in deionized water and ethanol, and washed several times. Finally, FISPAEK was obtained after vacuum drying at 80°C for 24 h.

1.3 Synthesis of Crosslinked PAEK Films

Polymer films with a thickness of 8-12 μm were prepared by solution casting. A polymer solution in NMP with a concentration of approximately 2 wt% was cast onto glass plates. After complete evaporation of the solvent at 70°C, the films were

annealed on the glass plates at 200°C for 4 h, during which the pendant allyl double bonds underwent thermal crosslinking. The films were then peeled from the glass plates. Au electrodes with a diameter of 10 mm and a thickness of 50 nm were deposited on both sides of each film for subsequent electrical measurements.

2. Characterization

Fourier transform infrared spectroscopy (FTIR) was performed using a Tensor 27 spectrometer in attenuated total reflection mode over the range of 500-4000 cm^{-1}. ^{1}H NMR spectra of the polymers were recorded on a Bruker AVANCE NEO 400 MHz spectrometer using deuterated chloroform ($CDCl_3$) as the solvent. Gel permeation chromatography (GPC) measurements were carried out on a Waters ACQUITY APC system at 30°C using THF as the eluent at a flow rate of 1.0 mL min^{-1}. UV-Vis absorption spectra of the polymer samples were measured using a PE Lambda 950 spectrophotometer. The mechanical properties of the PAEK-based dielectric films were measured using a universal testing machine. Dynamic mechanical analysis (DMA) was performed on a Netzsch DMA242E instrument to determine the glass transition temperature (T_g) and modulus. Dielectric properties were measured using a Novocontrol Concept 80 broadband dielectric spectrometer over a frequency range of 10^{-1}-10^{6} Hz at room temperature and 200°C. Films with a diameter of 10 mm and Au electrodes deposited on both surfaces were used for the measurements. Surface potential was characterized using an SPM-9700HT Kelvin probe force microscope (KPFM). An 8 V bias was applied to a film specimen for 10 min. After removal of the bias, the surface potential was monitored at 2 min intervals. The charging and discharging behavior of the dielectric films was evaluated using a laboratory-built capacitor charge-discharge system. Electric displacement-electric field (*D-E*) loops and leakage current were measured at 10 Hz using a FERRO20B ferroelectric test system. Au electrodes with an area of 0.069 cm^2 were deposited on both sides of the film samples. Breakdown strength was evaluated using an automatic

voltage-withstand tester (RK2674B) under a DC voltage ramp rate of 500 $V \cdot s^{-1}$ according to ASTM D149.

Gaseous products generated during thermal decomposition were analyzed using an STA8000-Spectrum 3-Clarus690 SQ8C TG-IR system under nitrogen. The samples were heated from 30 to 800°C at a rate of 20°C·min^{-1}. Field-emission scanning electron microscopy (SEM) images and EDS elemental maps were recorded using a Gemini SEM 500 microscope. Electrical self-healing images were obtained using a Zeiss Axio Scope A1 metallographic microscope.

3. Calculation Simulation

The structures were fully optimized using the hybrid DFT (density functional theory)-B3LYP function with the Gaussian 16 program package. while the 6-31G(d) basis sets were adopted for H, C, N, O and F atoms. Grimme's dispersion correction with Becke-Johnson damping was applied. TD-DFT was appilied at CAM-B3LYP/6-311g** level. Wave-function analyses were performed with multiwfn 3.8 (dev). All images were visualized by VMD.

The ODI value for orbital i is calculated by the following equation:

$$ODI_i = 0.01 \times \sum_A (\Theta_{A,i})^2 \qquad \text{(S1)}$$

where $\Theta_{A,i}$ represents the contribution of atom A to orbital i. A smaller value indicates more orbital delocalization. The value range is (0, 100].

4. Supplementary Figures

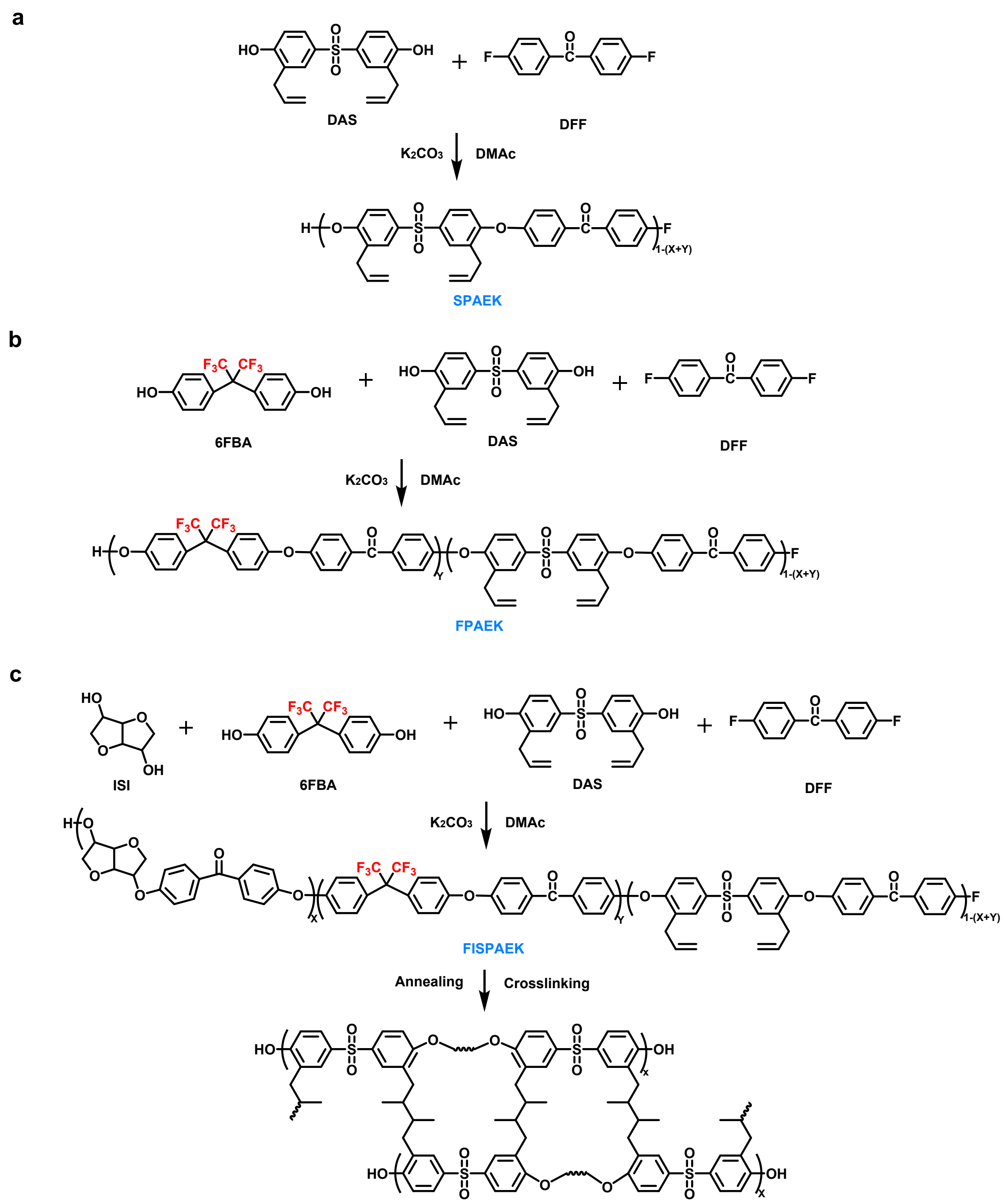

Figure S1 a) Synthetic route of SPAEK. b) Synthetic route of FPAEK. c) Synthetic route of FISPAEK.

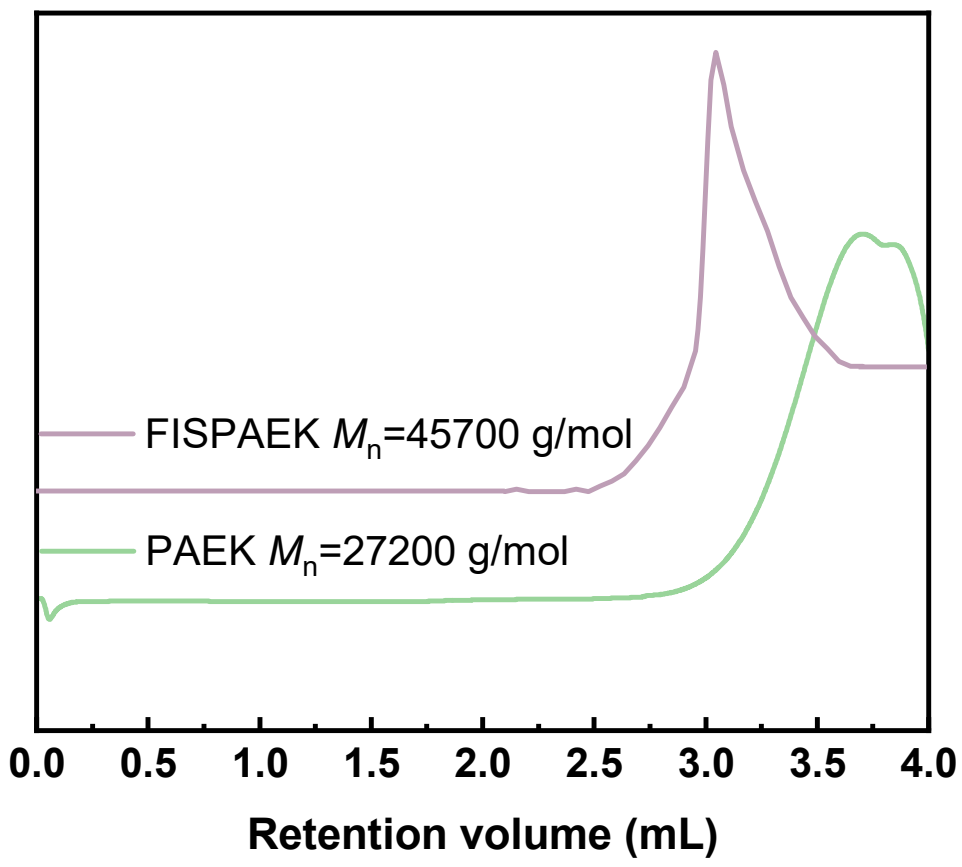

Figure S2 Number-average molecular weights of PAEK and FISPAEK.

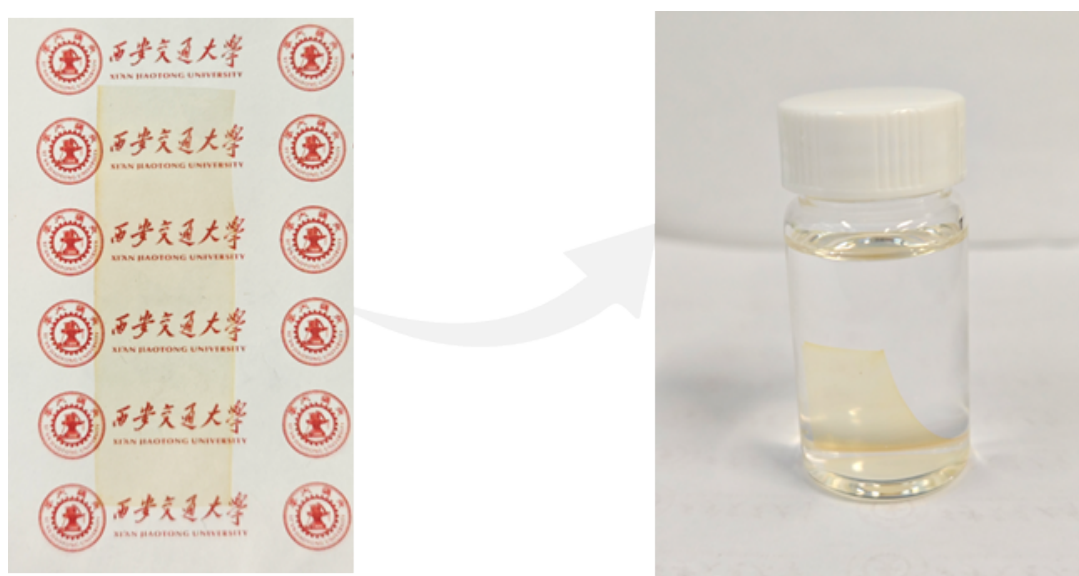

Figure S3 Photograph of the FISPAEK film in solvent after 60 days.

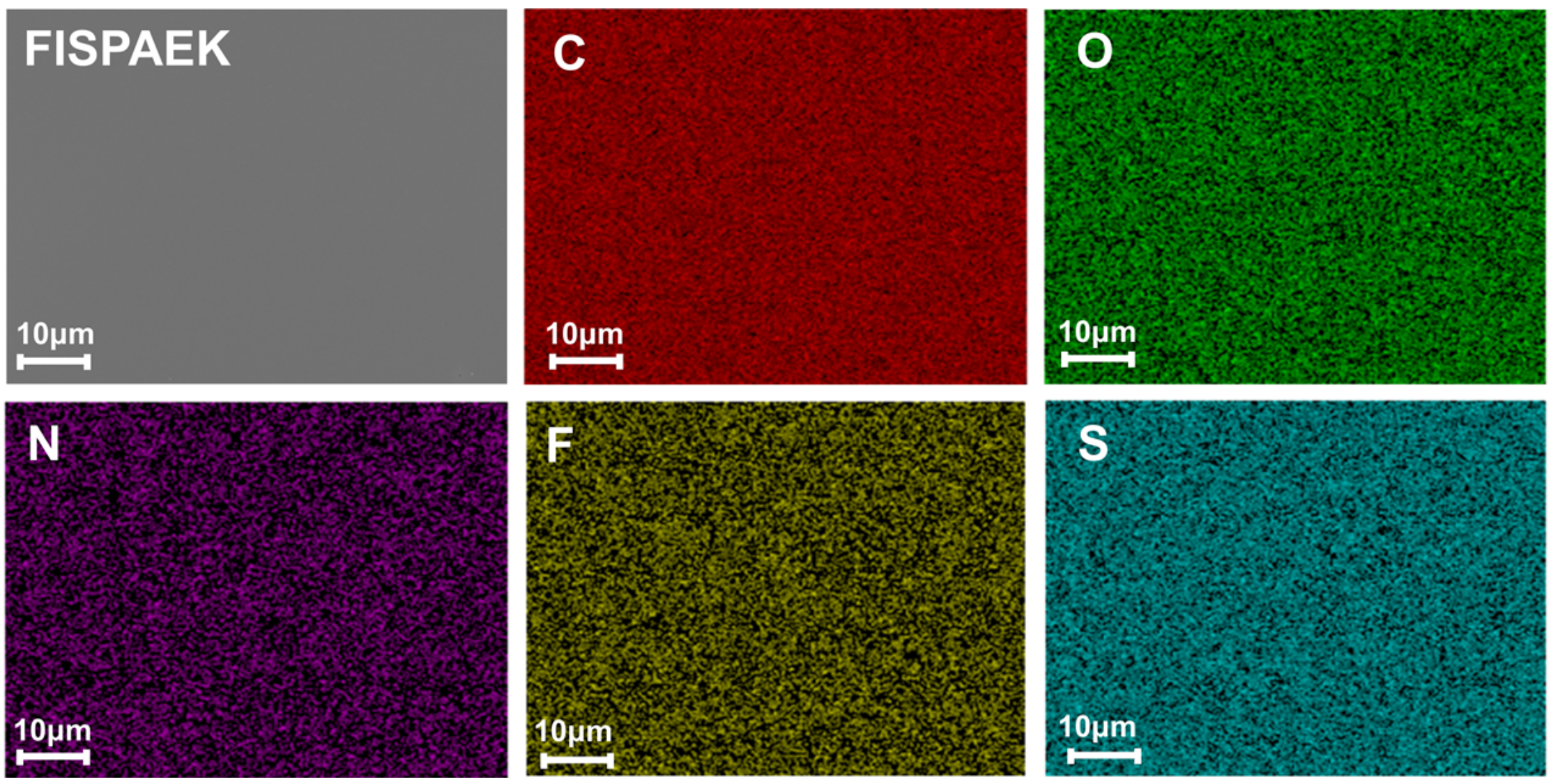

Figure S4 SEM image and surface elemental EDS maps of the FISPAEK film.

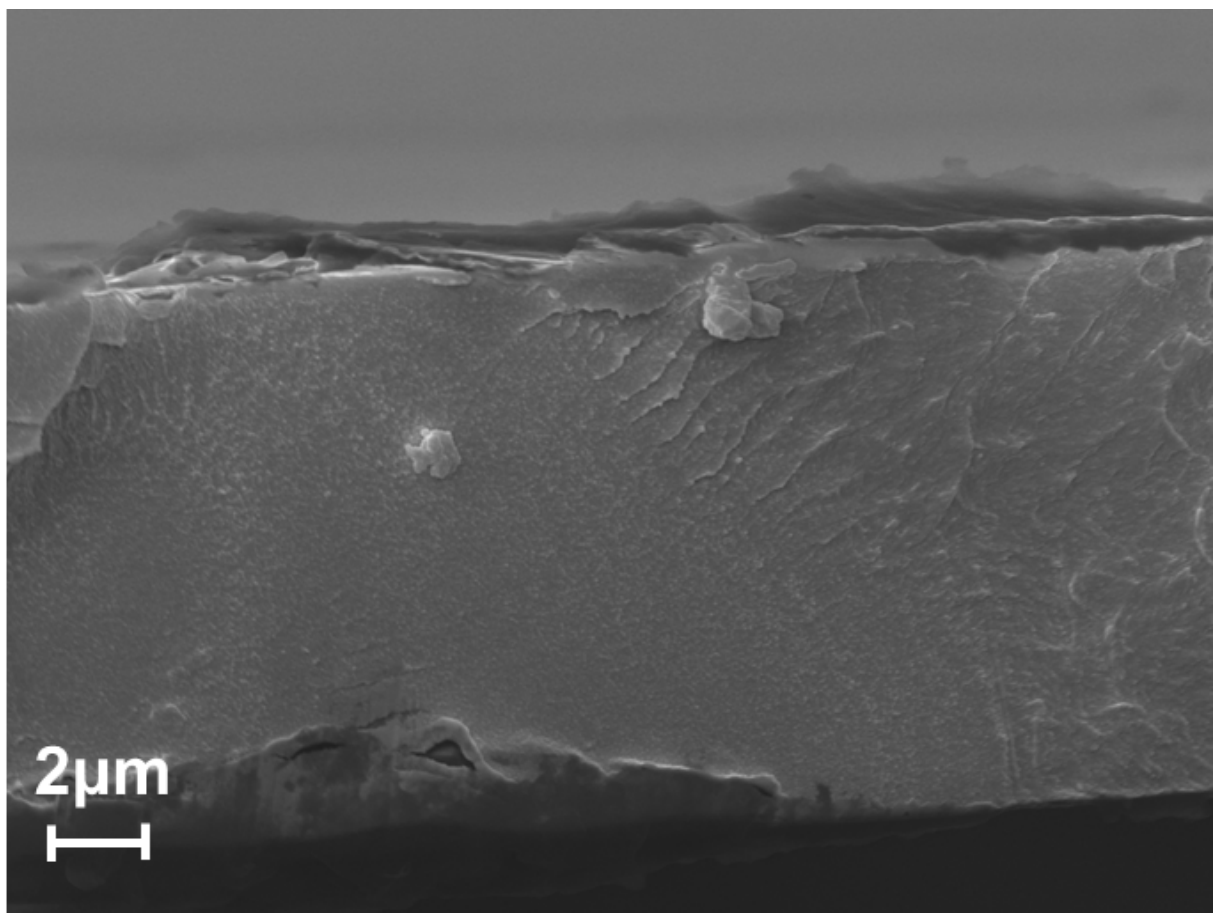

Figure S5 Cross-sectional SEM image of the FISPAEK film.

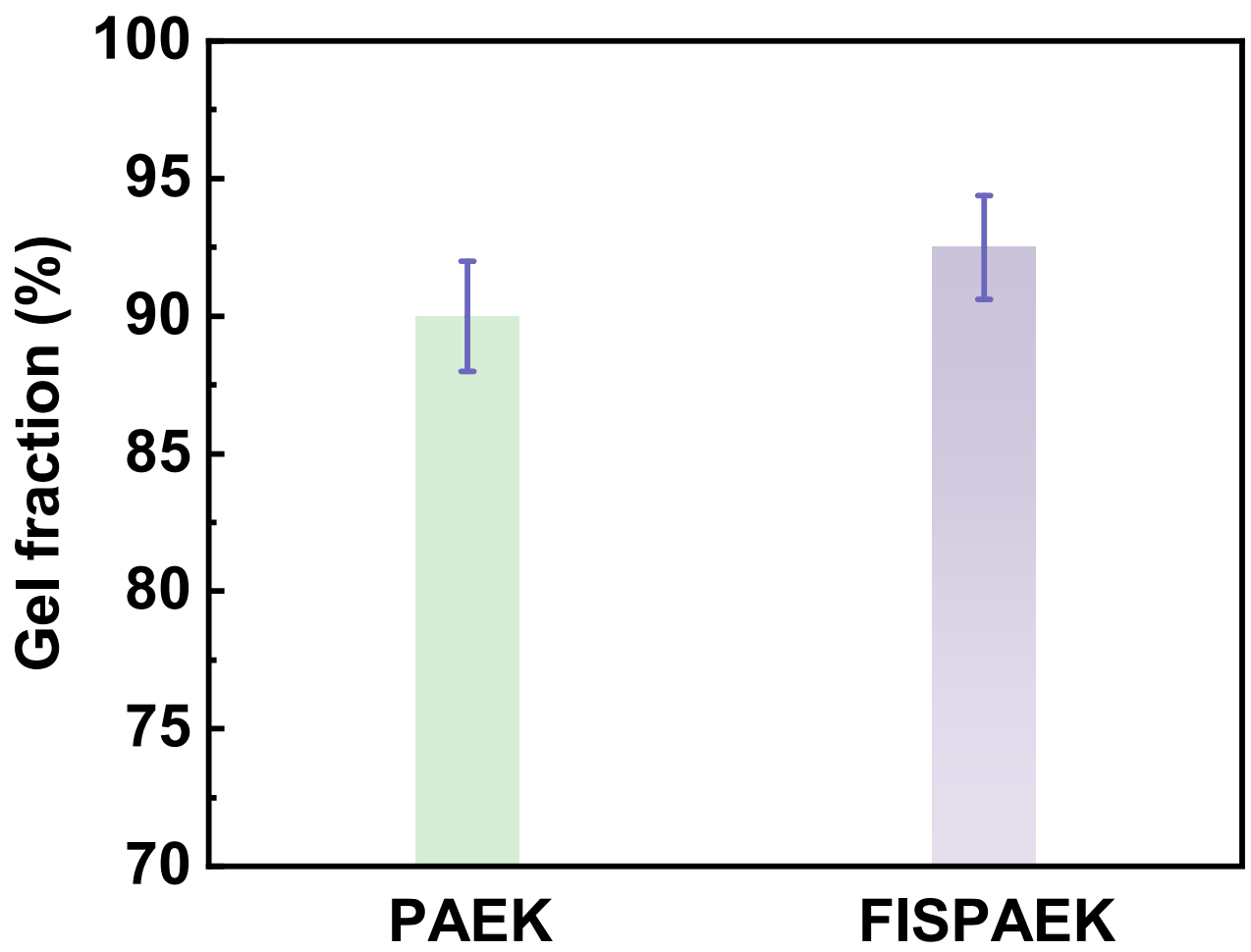

Figure S6 Gel fraction of PAEK and FISPAEK and the corresponding degrees of crosslinking.

To evaluate the gel fraction and network formation of the heat-treated films, equilibrium swelling experiments were carried out in N,N-dimethylacetamide (DMAc). The heat-treated films were first dried to constant weight and weighed to obtain the initial dry mass ($W_{initial}$). The films were then immersed in DMAc for 12 h until swelling equilibrium was reached. Three parallel samples were tested for each composition. After swelling, the films were removed from the solvent and gently wiped with filter paper to remove surface solvent. The samples were subsequently dried to constant weight again to obtain the dry mass of the insoluble fraction after

extraction (W_{after}).

The gel fraction was used as an indicator to evaluate the formation of the crosslinked network and the relative network compactness of the films.

The gel fraction (G_f) was calculated according to:

$$G_f(\%) = \frac{W_{after}}{W_{initial}} \times 100\%$$

where $W_{initial}$is the initial dry mass of the film before swelling and $W_{after}$is the dry mass of the insoluble fraction after solvent extraction. A higher gel fraction indicates a larger insoluble network fraction, suggesting a more developed and compact crosslinked network.

As shown in Figure S6, FISPAEK exhibits a higher gel fraction than PAEK, indicating the formation of a more developed crosslinked network and enhanced network compactness.

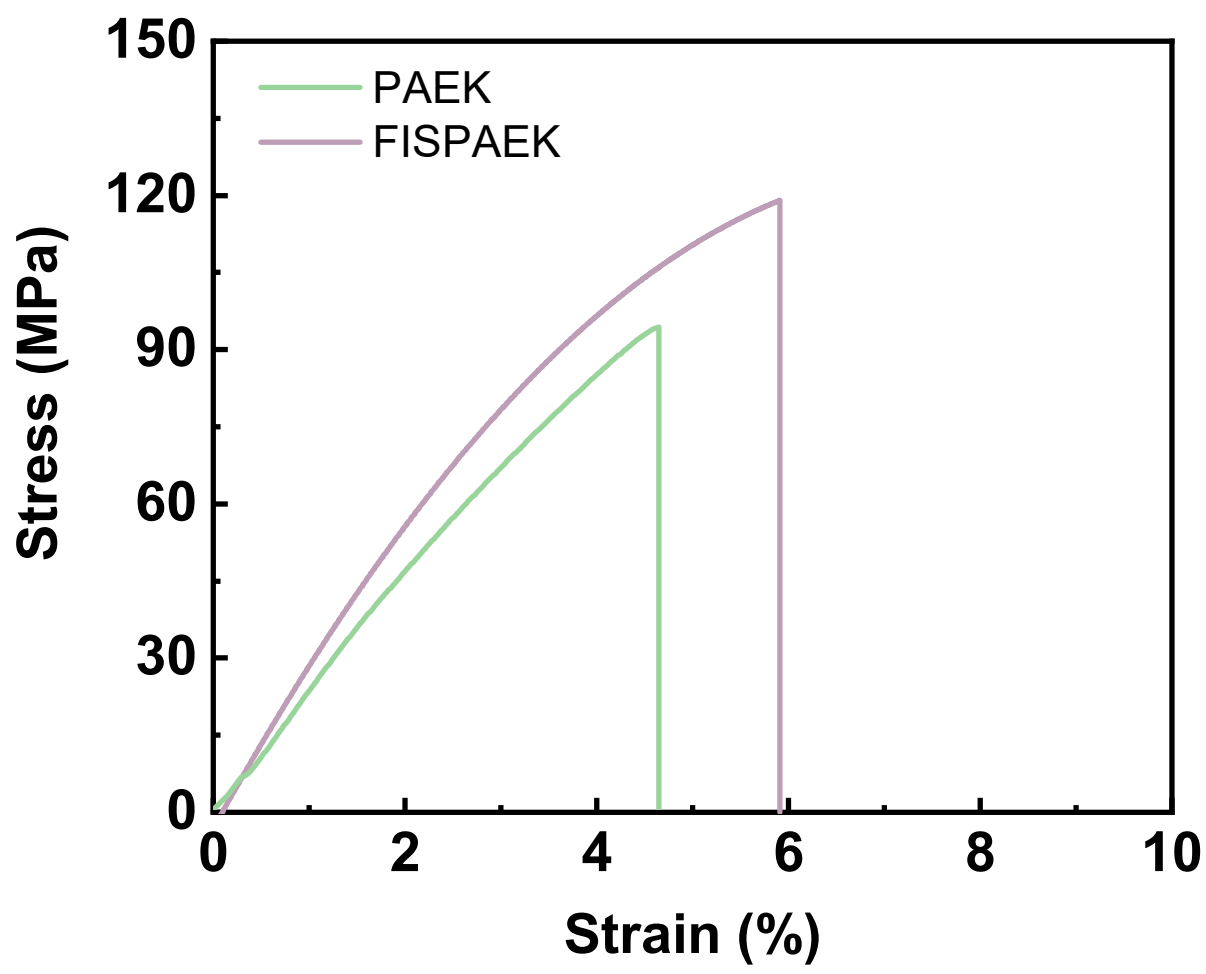

Figure S7 Stress-strain curves of PAEK and FISPAEK films.

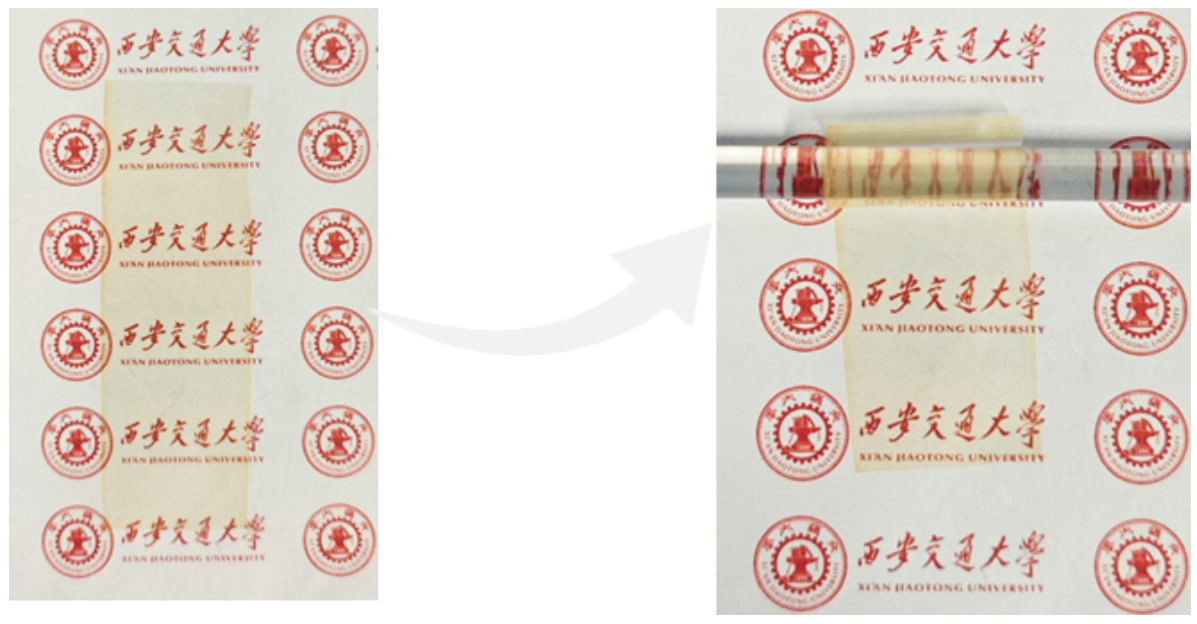

Figure S8 Photograph of the FISPAEK film wound around a glass rod, demonstrating its flexibility.

Table S1 Comparison of the compositions of different PAEKs.

Sample	IS:6F:DAS
FISPAEK	1:1:4
FISPAEK-IS	1.5:0.5:4
FISPAEK-DAS	0.5:1:4.5
FISPAEK-6F	1:1.5:3.5

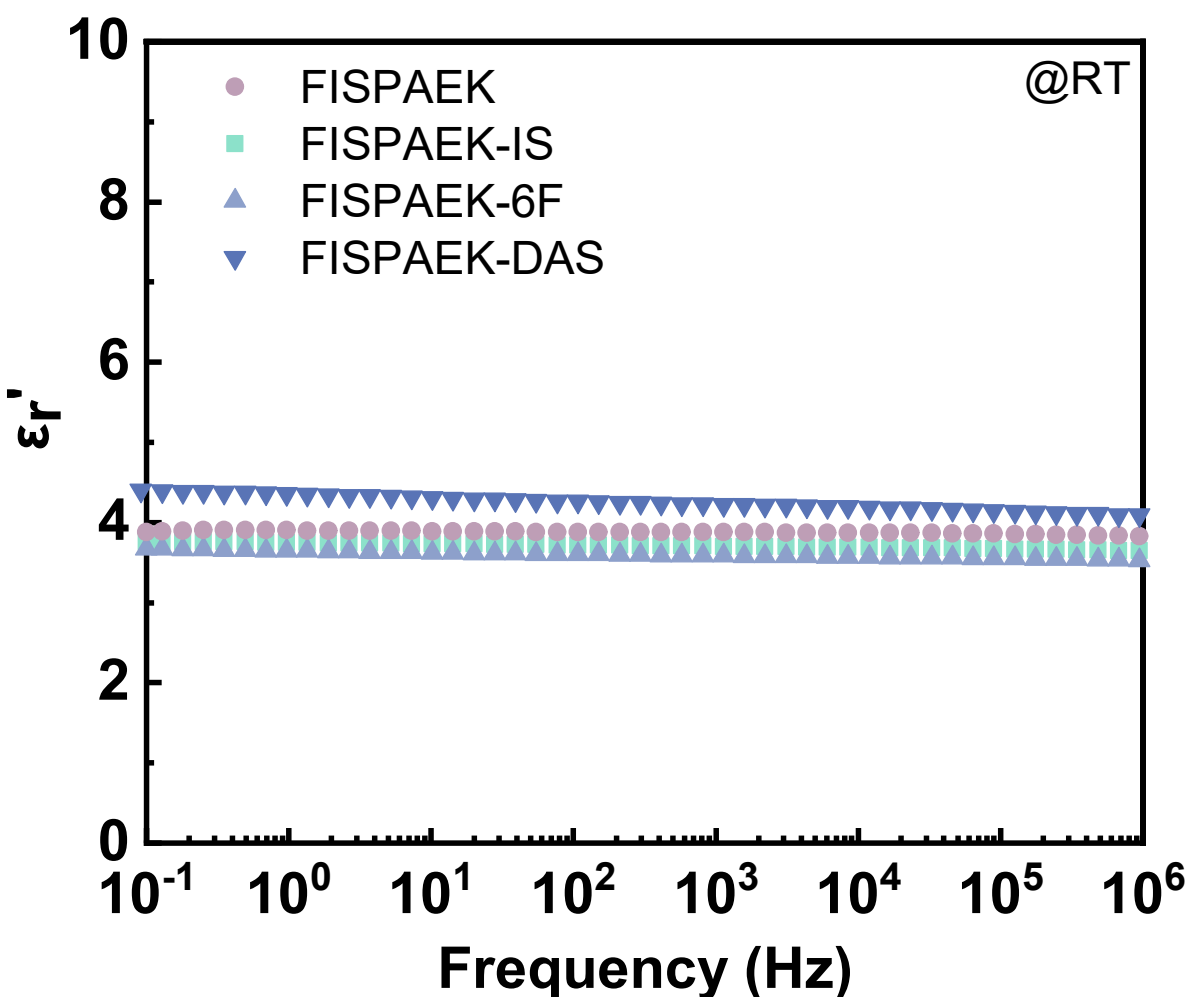

Figure S9 Frequency-dependent dielectric constant of different PAEKs at room temperature.

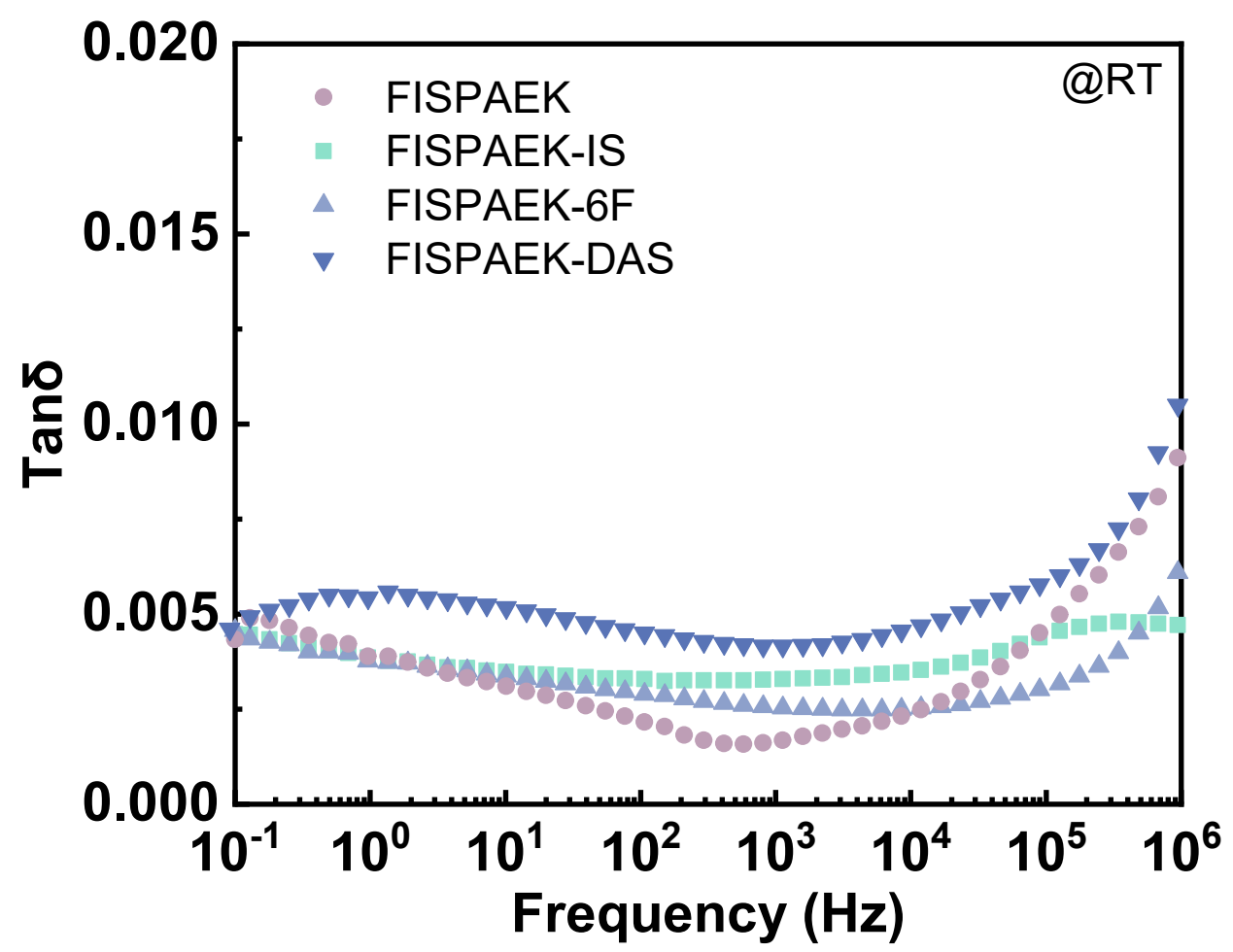

Figure S10 Frequency-dependent dielectric loss of different PAEKs at room temperature.

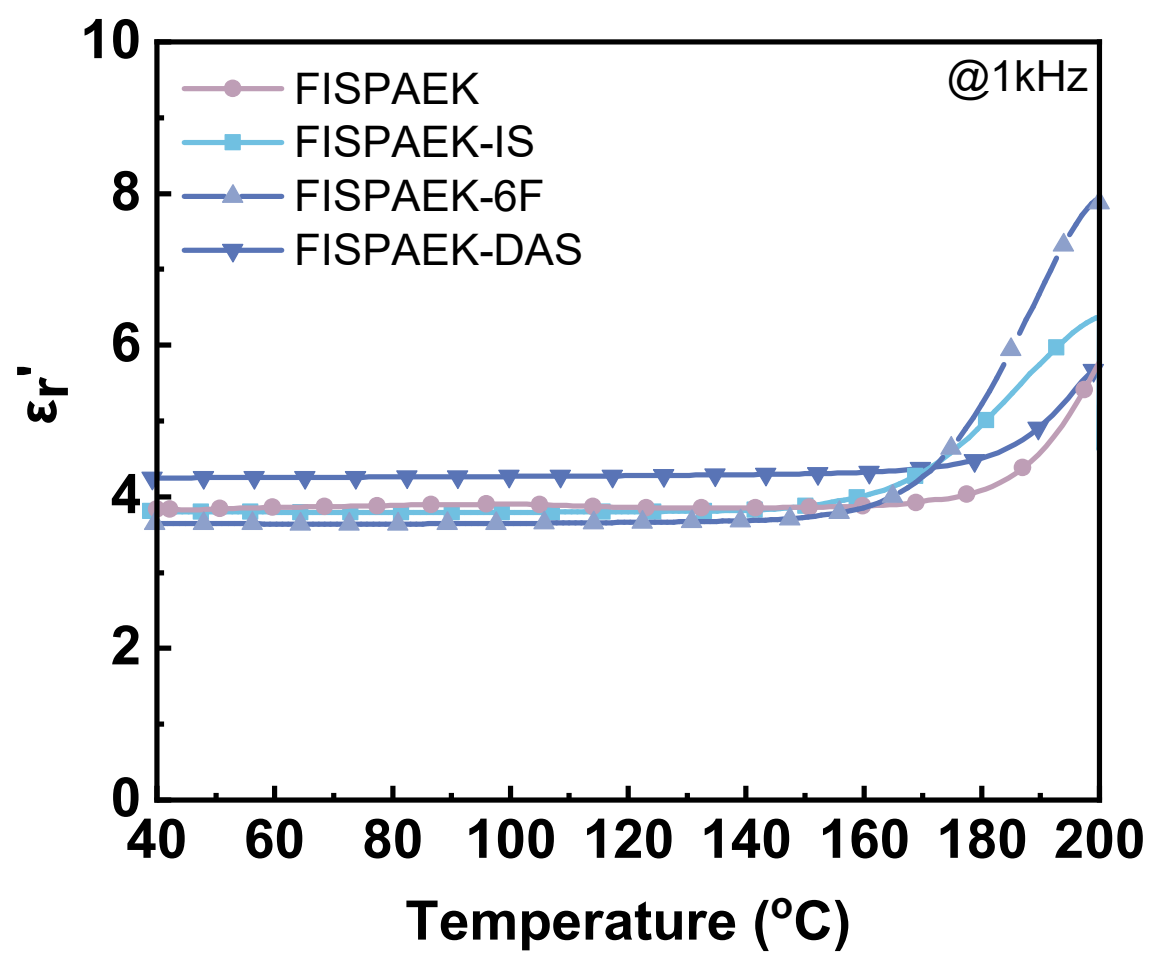

Figure S11 Temperature-dependent dielectric constant of different PAEKs at 1 kHz.

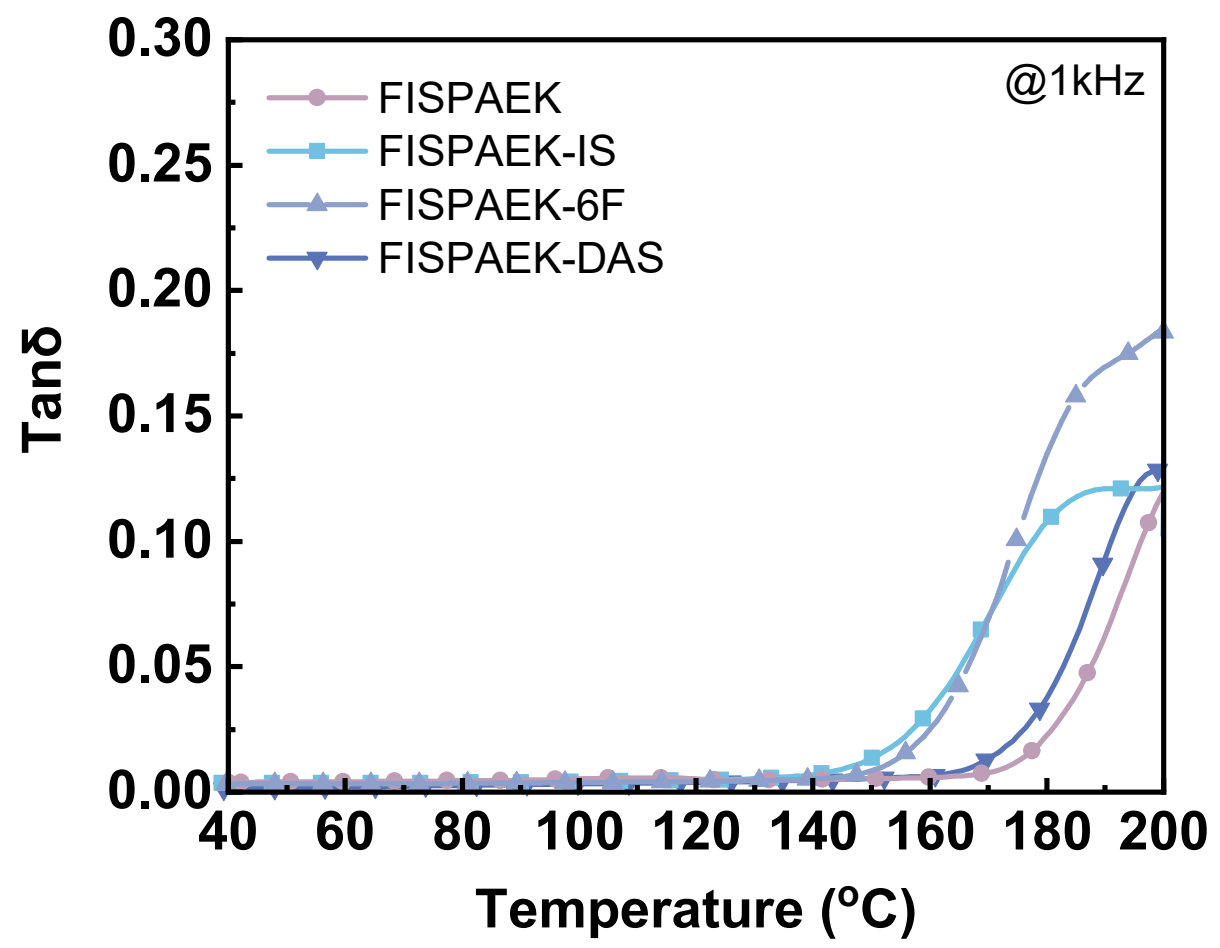

Figure S12 Temperature-dependent dielectric loss of different PAEKs at 1 kHz.

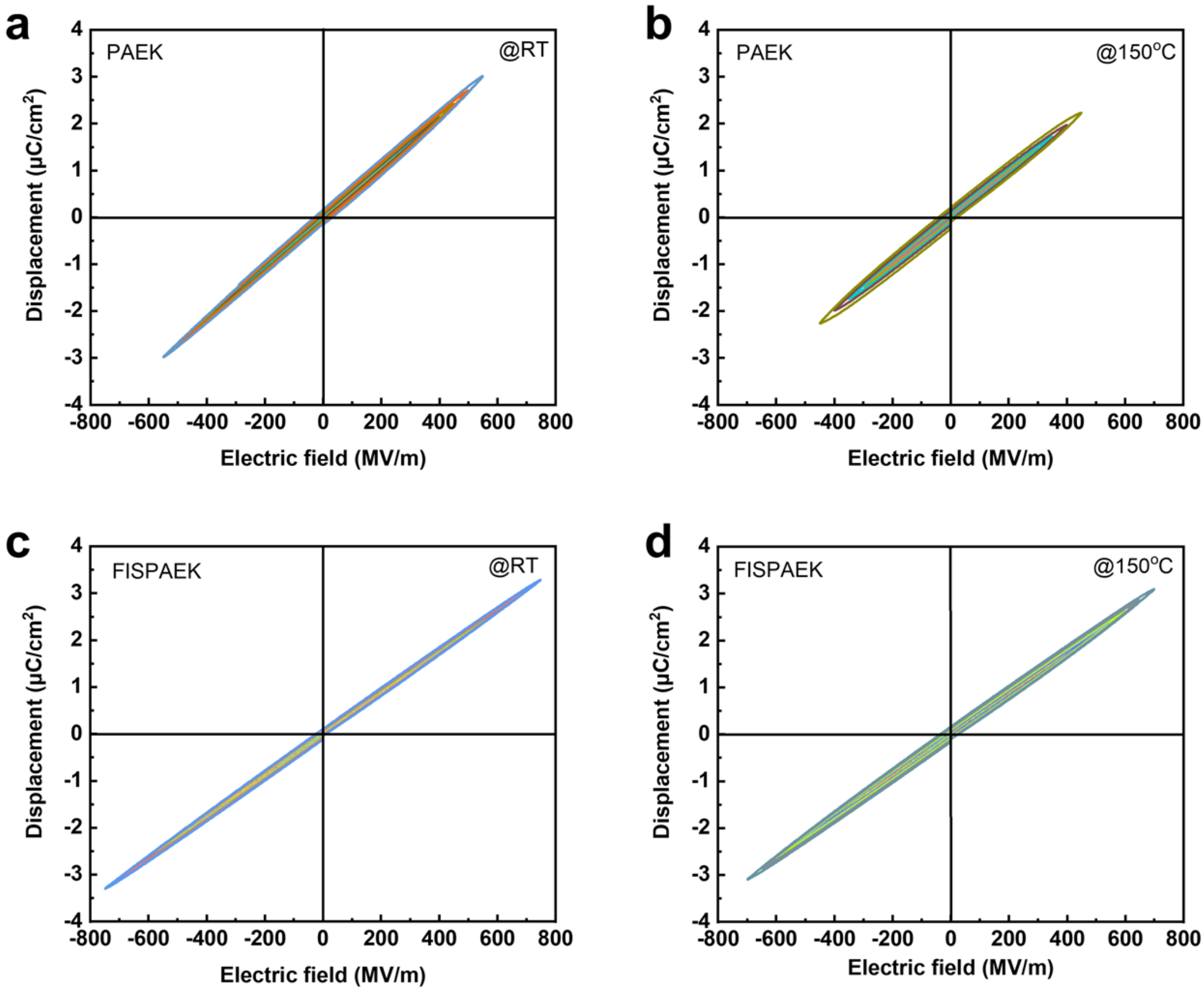

Figure S13 *D-E* loops of PAEK and FISPAEK at room temperature and 150°C.

Table S2 Comparison chart of U_e and η.

Material	Temperature (°C)	U_e (J·cm^{-3})	η (%)
**c-P(AEK-BBP)**	150	5.5	93
**BT-BCB@DPAES**	150	4.5	84
**PEI/PCBM**	150	3.9	78
**PAEN-CN**	150	3.8	84
**PEPA-PEI**	150	3.7	96
**PEEK-CN**	150	3.4	78
**PI-PAA-BNNS**	150	3.0	52
**PEKK**	150	2.8	61
**PEEU**	150	2.9	75

**PPENK**	150	2.1	69
**PTFE**	150	2.7	90
**PEKK**	150	2.4	52
**PPEK**	150	2.5	70
**p-POCINB**	150	4.7	90
**PPES**	150	3.7	60
**PEI**	150	1.6	39
**PC**	150	1.3	47
**PET**	150	0.9	57
**PEN**	150	1.0	77
**PPS**	150	0.4	40
**PI**	150	0.8	34
**PEI/4-NB**	150	5.2	90
**c-P（AEK–BBP）**	150	5.3	91
**PPO/PS-75/25**	150	5.5	92.3
**PEEU**	150	2.9	75
**PEIs/PI/PEIs**	150	3.2	85
**c-BCB/BNNS**	150	2.0	90
**BN-BCB/c-DPAEK**	150	3.8	90
**PEI/PCBM**	150	4.5	90
**this work 2**	150	6.9	90

Table S4. Characteristic TG-IR absorption bands and assignments of the gaseous decomposition products of FISPAEK.

Characteristic band (cm^{-1})	Vibrational assignment	Identified gaseous product
668	Aromatic C-H out-of-plane bending, δ(C-H)	Benzene
927, 1106	Fingerprint vibrations of fluorinated aromatic species; C-F stretching mainly contributes to the band near 1106 cm^{-1}	Fluorobenzene (C_6H_5F)
1151, 1339	Symmetric and asymmetric S-O stretching	SO_2
1509	Aromatic ring C=C stretching, ν(C=C)	Aromatic species (arene)
1701	Carbonyl C=O stretching, ν(C=O)	Ketone-containing volatile species
2358	Asymmetric O=C=O stretching	CO_2
≈3567	O-H stretching, ν(O-H)	Aromatic hydroxyl-containing species (Ar-OH)
3947-3976	H-F stretching, ν(H-F)	HF
